# P-2030. Remdesivir and Obeldesivir Retain Potent Activity Against SARS-CoV-2 Omicron Variants

**DOI:** 10.1093/ofid/ofae631.2186

**Published:** 2025-01-29

**Authors:** Lauren Rodriguez, J Lizbeth Reyes Zamora, Dong Han, Nadine Peinovich, Clarissa Martinez, Pui Yan Ho, Jiani Li, Thomas Aeschbacher, Andy Pekosz, John P Bilello, Jason K Perry, Charlotte Hedskog

**Affiliations:** Gilead Sciences, Inc., Foster City, CA; Gilead Sciences, Inc., Foster City, CA; Gilead Sciences, Inc., Foster City, CA; Gilead Sciences, Inc., Foster City, CA; Gilead Sciences, Inc., Foster City, CA; Gilead Sciences, Inc., Foster City, CA; Gilead Sciences, Inc., Foster City, CA; Gilead Sciences, Inc., Foster City, CA; Johns Hopkins University, Baltimore, Maryland; Gilead Sciences, Inc., Foster City, CA; Gilead Sciences, Inc., Foster City, CA; Gilead Sciences, Inc., Foster City, CA

## Abstract

**Background:**

Remdesivir (RDV), a nucleotide analog prodrug that targets the viral RNA-dependent RNA polymerase, Nsp12, is approved to treat COVID-19 in hospitalized and nonhospitalized patients. Obeldesivir (ODV), an oral mono-5’-isobutyryl ester prodrug, is metabolized into the same active triphosphate as RDV. The antiviral activity of RDV and ODV against previous Omicron subvariants (BA.1 to XBF) was maintained with respect to the ancestral WA1 strain. Here, RDV and ODV antiviral activity data against recent Omicron subvariants (XBB.2.3.2, EG.5.1, EG.1.2, BA.2.86, XBC.1.6, HK.3, and JN.1) are reported using clinical isolates and site directed mutants (SDMs) in replicons bearing Nsp12 substitutions observed in these subvariants.

**Methods:**

The prevalence of Nsp12 substitutions in Omicron subvariants was assessed using *Global Initiative on Sharing Avian Influenza Data* (GISAID) EpiCoV database sequences. Structures of identified substitutions were analyzed using a previously established cryo-electron microscopy-based model of the replication-transcription complex. Antiviral activity (half-maximal effective concentration [EC_50_]) of RDV and ODV against subvariant clinical isolates was assessed by nucleoprotein ELISA in A549-hACE2-TMPRSS2 cells and by SDMs in a replicon system.

**Results:**

Genomic analysis of >2.5 million Omicron subvariant sequences revealed unique substitutions in Nsp12 vs WA1. Two new defining mutations (≥75% of sequences; D63N [HK.3] and G823insD [XBC.1.6]) were found compared with earlier Omicron subvariants. Less prevalent substitutions (1.0% to 15.9%; T26I, D40G, T85I, I171V, Y175H, I223M, T225I, D258N, Y289H, D303N, T394M, P461S, V476A, M666I, T803I, and V848I) were also observed; none had direct interaction with the incoming nucleotide triphosphate or the viral RNA. Phenotyping of individual substitutions using SDMs showed no loss of RDV or ODV susceptibility (≤1.60-fold change). Phenotyping of clinical isolates of XBB.2.3.2, EG.5.1, EG.1.2, BA.2.86, XBC.1.6, HK.3, and JN.1 indicated no change of RDV or ODV in vitro antiviral activity (< 1.05-fold change).

**Conclusion:**

RDV and ODV retained potent in vitro antiviral activity against all tested Omicron subvariants and Nsp12 substitutions with potencies comparable to reference strains.

**Disclosures:**

Lauren Rodriguez, PhD, Gilead Sciences, Inc.: Employee|Gilead Sciences, Inc.: Stocks/Bonds (Public Company) J. Lizbeth Reyes Zamora, PhD, Gilead Sciences, Inc.: Employee|Gilead Sciences, Inc.: Stocks/Bonds (Public Company) Dong Han, MS, Gilead Sciences, Inc.: Employee|Gilead Sciences, Inc.: Stocks/Bonds (Public Company) Nadine Peinovich, MPH, Gilead Sciences, Inc.: Employee|Gilead Sciences, Inc.: Stocks/Bonds (Public Company) Clarissa Martinez, MPH, Gilead Sciences, Inc.: Employee|Gilead Sciences, Inc.: Stocks/Bonds (Public Company) Pui Yan Ho, PhD, Gilead Sciences, Inc.: Employee|Gilead Sciences, Inc.: Stocks/Bonds (Public Company) Jiani Li, PhD, Gilead Sciences, Inc.: Employee|Gilead Sciences, Inc.: Stocks/Bonds (Public Company) Thomas Aeschbacher, PhD, Gilead Sciences, Inc.: Employee|Gilead Sciences, Inc.: Stocks/Bonds (Public Company) John P. Bilello, PhD, Gilead Sciences, Inc.: Employee|Gilead Sciences, Inc.: Stocks/Bonds (Public Company) Jason K. Perry, PhD, Gilead Sciences, Inc.: Employee|Gilead Sciences, Inc.: Stocks/Bonds (Public Company) Charlotte Hedskog, PhD, Gilead Sciences, Inc.: Employee|Gilead Sciences, Inc.: Stocks/Bonds (Public Company)

